# Evaluation of diagnostic accuracy and dimensional measurements by using CBCT in mandibular first molars

**DOI:** 10.4317/jced.52570

**Published:** 2016-02-01

**Authors:** Saeed Asgary, Sima Nikneshan, Alireza Akbarzadeh-Bagheban, Naghmeh Emadi

**Affiliations:** 1 Iranian Center for Endodontic Research, Research Instituteof Dental Sciences, Shahid Beheshti University of Medical Sciences, Tehran, Iran; 2 Department of Oral and Maxillofacial Radiology, Dental School, Shahid Beheshti University of Medical Sciences, Tehran, Iran; 3 Department of Basic Sciences, School of Rehabilitation Sciences, Shahid Beheshti University of Medical Sciences, Tehran, Iran; 4 Dental Research Center, Research Institute of Dental Sciences, Shahid Beheshti University of Medical Sciences, Tehran, Iran

## Abstract

**Background:**

This study aimed to assess the diagnostic accuracy of cone beam computed tomography (CBCT) and quantitatively evaluate the morphology of mandibular first molars using CBCT.

**Material and Methods:**

Twenty-four double-rooted mandibular first molars were evaluated by NewTom VGi CBCT. The distance from the furcation and apex to the cementoenamel junction (CEJ), diameter and thickness of canal walls, the buccolingual (BL) to mesiodistal (MD) ratio (ΔD), prevalence of oval canals at different sections and taper of the canals were all determined. In order to assess the diagnostic accuracy of CBCT, distance from the furcation and apex to the CEJ and thickness of canal walls at the CEJ and apex were compared with the gold standard values (caliper and stereomicroscope). Statistical analyses were carried out using intraclass correlation coefficient (ICC), paired t-test and repeated measures ANOVA.

**Results:**

A high correlation existed between the CBCT and gold standard measurements (*P*<0.001). In dimensional measurements, length of mesial root was higher than the distal root and lingual furcation was farther from the CEJ than the buccal furcation (*P*<0.001). An important finding of this study was the mesiodistal taper of the mesiobuccal (MB) and mesiolingual (ML) canals; which was equal to 0.02.

**Conclusions:**

CBCT has acceptable diagnostic accuracy for measurement of canal wall thickness. Cleaning and shaping of the canals should be performed based on the unique anatomy of the respective canal; which necessitates the use of advanced imaging techniques for thorough assessment of root canal anatomy in a clinical setting.

** Key words:**Permanent mandibular first molar, accuracy, cone-beam computed tomography, dimensional measurement.

## Introduction

Adequate knowledge about the root canal anatomy is a necessary prerequisite for a successful root canal treatment (RCT) (1). Root canal is composed of high-contrast tissues. Tachibana and Matsumoto were the first to use tomography to explore the root canal system. However, due to the poor resolution of medical computed tomography it cannot thoroughly evaluate the root canal details (2). Recently, micro-computed tomography (μ-CT) has been used as a suitable tool for the 3D reconstruction of internal and external tooth morphology due to its high resolution for root canal studies (3).

Many studies have successfully used μ-CT for quantitative and qualitative assessment of the root canal system under *in-vitro* conditions (4). However, this imaging modality is time consuming and not easily accessible for use in the office setting. In contrast to conventional CT, CBCT provides lower radiation dose and faster acquisition time (5). With a limited field of view, optimal spatial resolution is achieved in all planes. One advantage of CBCT is multiplanar reformation 3D surface rendering (6). CBCT has been used for diagnosis, treatment planning and pre-surgical assessment in many dental fields. However, at present, CBCT has limited application for quantitative and qualitative study of the root canal dimensions. In contrast to μ-CT, the potential of CBCT for detailed evaluation of root canal system has yet to be evaluated.

This study aimed to assess the diagnostic accuracy of CBCT and quantitatively evaluate the morphology of mandibular first molars using CBCT.

## Material and Methods

**Figure 1:**
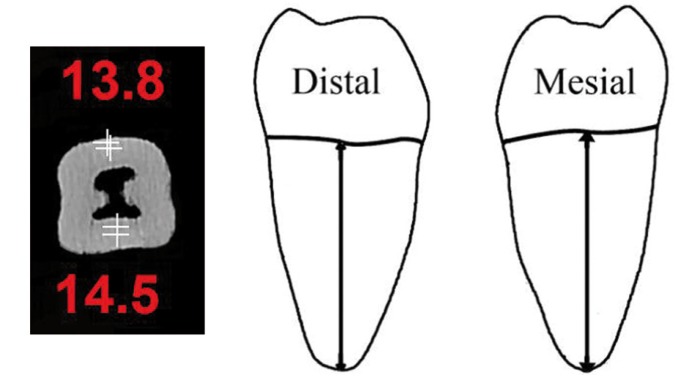
CBCT scan and schematic view of mesial and distal root length measurements.

**Figure 2:**
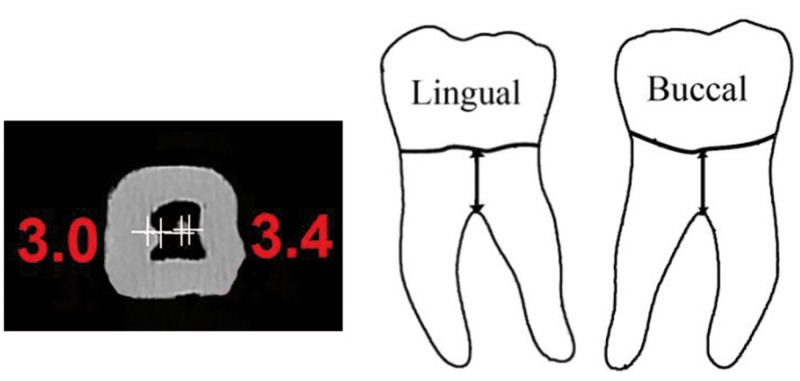
CBCT scan and schematic view of the distance from the buccal and lingual furcations to the CEJ.

Preparation of specimens and CBCT measurements: Teeth crowns were cut at the CEJ and the roots were embedded in dental putty (Zhermack, Italy) blocks. High-resolution CBCT radiographs were obtained of each tooth using New Tom VGi CBCT (Verona, Italy). The exposure settings were 6×6 field of view, 0.1 mm voxel size, 110 kVp and 0.56 mA. Following image reconstruction by NNT Viewer software, linear measurements were made by displaying the images on a Philips monitor with 1024×1280 pixels resolution and 32 bit color depth. All measurements were made by an oral and maxillofacial radiologist in triplicate. The observers were allowed to adjust the contrast, resolution and brightness of images for more accurate measurements. Measurements were made at the following sites:

1. Root area: Vertical distance from the apex to the proximal CEJ along the long axis of the teeth in the mesial and distal roots (Fig. 1).

2. Furcation area: Vertical distance from the lowest furcation surface to the proximal CEJ along the long axis of the tooth for buccal and lingual furcations (Fig. 2).

It should be noted that in order to determine the diagnostic accuracy of CBCT in the apex and furcation areas, the actual measurements were made by a highly accurate caliper (Mitutoyo, Japan) and considered as the gold standard.

**Figure 3:**
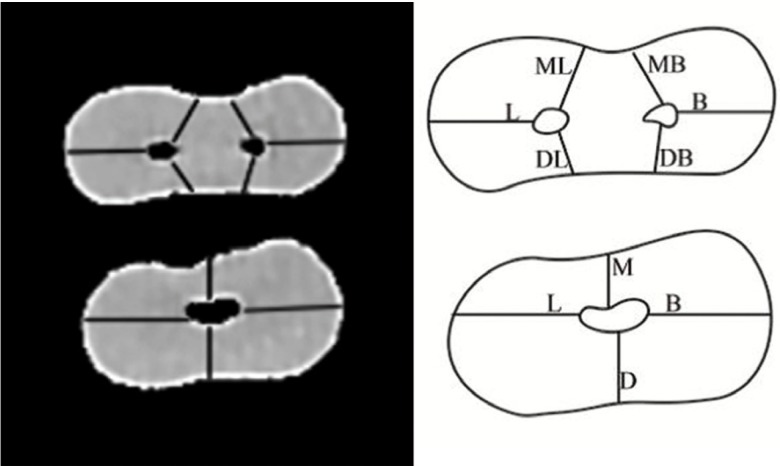
CBCT scan and schematic view of the canal wall thickness in the mesial and distal roots.

3. Root canal: Wall thickness and diameter of the canal were measured on CBCT radiographs in a maximum of 12 sections with 1mm intervals from the furcation to the apex as follows:

-The canal wall thickness in buccal, lingual, MB, ML, distobuccal (DB) and distolingual (DL) dimensions of the mesial root and the canal wall thickness in the buccal, lingual, mesial and distal dimensions of the distal root were measured. Measurement of canal wall thickness in mesial and distal dimensions was repeated several times and the operator tried to choose the shortest distance. For buccal and lingual walls, the longest distance was chosen (Fig. 3).

-Canal diameter was measured in the MB, ML and distal roots in BL and MD dimensions.

Considering the above-mentioned measurements, the following calculations were done:

The buccolingual to mesiodistal ratio (ΔD) was calculated. ΔD<2 indicated a round and ΔD>2 indicated an oval canal.

-For calculation of canal taper, canal diameter at the furcation area was subtracted from the canal diameter at the apex and divided by the longitudinal furcation-apex distance.

*In order to confirm the accuracy of canal wall thickness by CBCT, 30 single-rooted teeth were used. Teeth crowns were cut at the CEJ and at 4mm distance from the apex. Dentin thickness at the CEJ and at 4mm distance from the apex was measured from the internal surface of the canal to the external root surface in buccal, lingual, mesial and distal directions using a stereomicroscope (Olympus, Japan) with ×12 magnification and considered as the gold standard. CBCT radiographs were then obtained from the teeth, and the canal wall thickness at the above-mentioned sections was measured and compared to the gold standard.

In addition to descriptive statistics namely the mean and standard deviation, ICC was applied for the comparison of CBCT and the gold standard measurements. Paired t-test and repeated measures ANOVA were also used for statistical analysis.

## Results

Twenty-five double-rooted mandibular first molars with two canals in the mesial and one canal in the distal root were evaluated. One tooth was excluded from the study due to having two canals in the distal root.

-Validity assessment.

ICC test showed a significant correlation between the CBCT and the gold standard measurements (*P*<0.001). The ICC values for the measurement of mesial root length, distal root length and distance from the buccal and lingual furcations to the CEJ were 0.995, 0.996, 0.988 and 0.982, respectively. The mean error using Dalber’s method in the mentioned sections was 0.087, 0.073, 0.103 and 0.108, respectively. Statistical analyses for the accuracy of canal wall thickness measurements at the CEJ and at the apex in all dimensions showed a significant correlation (*P*<0.001) with ICC>0.996 (0.998 at the CEJ and 0.996 at 4mm distance from the apex). The mean error using Dalber’s method in the mentioned surfaces was 0.053 and 0.062mm, respectively.

-Measurement of canal dimensions:

The distance from the apex to the CEJ: The mean mesial and distal root length from the apex to the CEJ is shown in  Table 1 indicating that the mesial root was 1mm longer than the distal root and this difference was statistically significant (*P*=0.000).

**Table 1:**
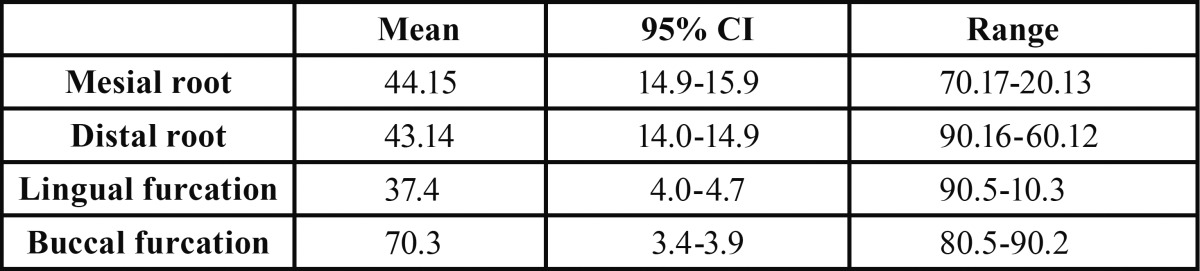
The mean, 95% confidence interval (95% CI) and range values for the distance from the apex (mesial and distal roots) to the CEJ and the distance from the furcation (buccal and lingual) to the CEJ (mm) (n=25).

The distance from the furcation to the CEJ: The mean distance from the furcation (buccal and lingual) to the CEJ is shown in  Table 1 indicating that the lingual furcation was farther from the CEJ than the buccal furcation and the difference in this respect was statistically significant (*P*=0.000).

Root canal area: A maximum of 12 sections within the furcation-apex distance were evaluated in each of the 24 first molars.  Table 2 summarizes the statistical indices of canal wall thickness in the mesial and distal roots. From the furcation towards the apex, a significant reduction in canal wall thickness was seen (*P*<0.001). Pairwise comparison of surfaces in the mesial root showed statistically significant differences (*P*<0.001) except for the buccal with lingual, MB with ML and DB with DL wall thicknesses. Within 4 mm distance from the furcation, the lowest canal wall thickness was seen at the DB and DL surfaces of the mesial root. DB and DL areas were not significantly different in this regard (*P*>0.05). On the other hand, DB and MB areas were significantly different (*P*<0.001) and in all sections, MB values were greater than DB values. This was also true for the DL and ML areas. With regard to distal root especially within 10mm distance from the furcation area, the lowest canal wall thickness was seen at the mesial wall. Pairwise comparison of buccal, lingual, mesial and distal walls of the distal root showed significant differences (*P*<0.05).

**Table 2:**
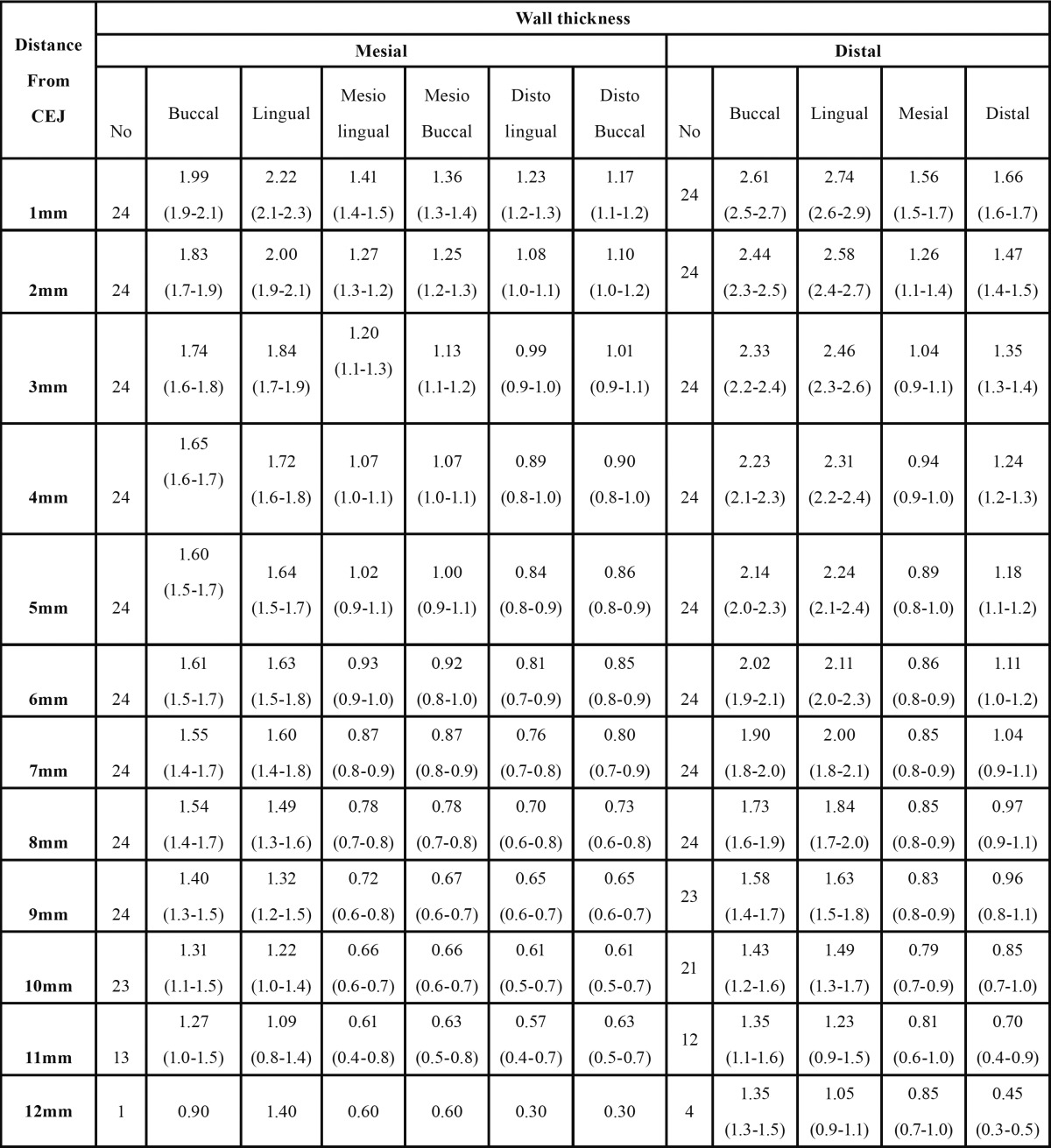
The mean (95% CI) canal wall thickness of the mesial and distal roots and distance from the furcation (mm).

 Table 3 shows statistical indices of canal diameter, BL to MD ratio (ΔD) and prevalence of oval canals in the mesial and distal roots. It appears that due to tapering, canal diameter decreases from the furcation towards the apex. However, as seen in  Table 3, in some sections the wall thickness values did not follow a regular sequential order from the furcation towards the apex; which is due to the anatomical variations and intracanal configurations. In our study, mesial root canals were Vertucci’s type IV in 18 and Vertucci’s type II in the remaining 6 teeth. The prevalence of oval canals in the distal root was over 90% within 5mm distance from the furcation area; whereas, this rate was less than 50% in the mesial root.

**Table 3:**
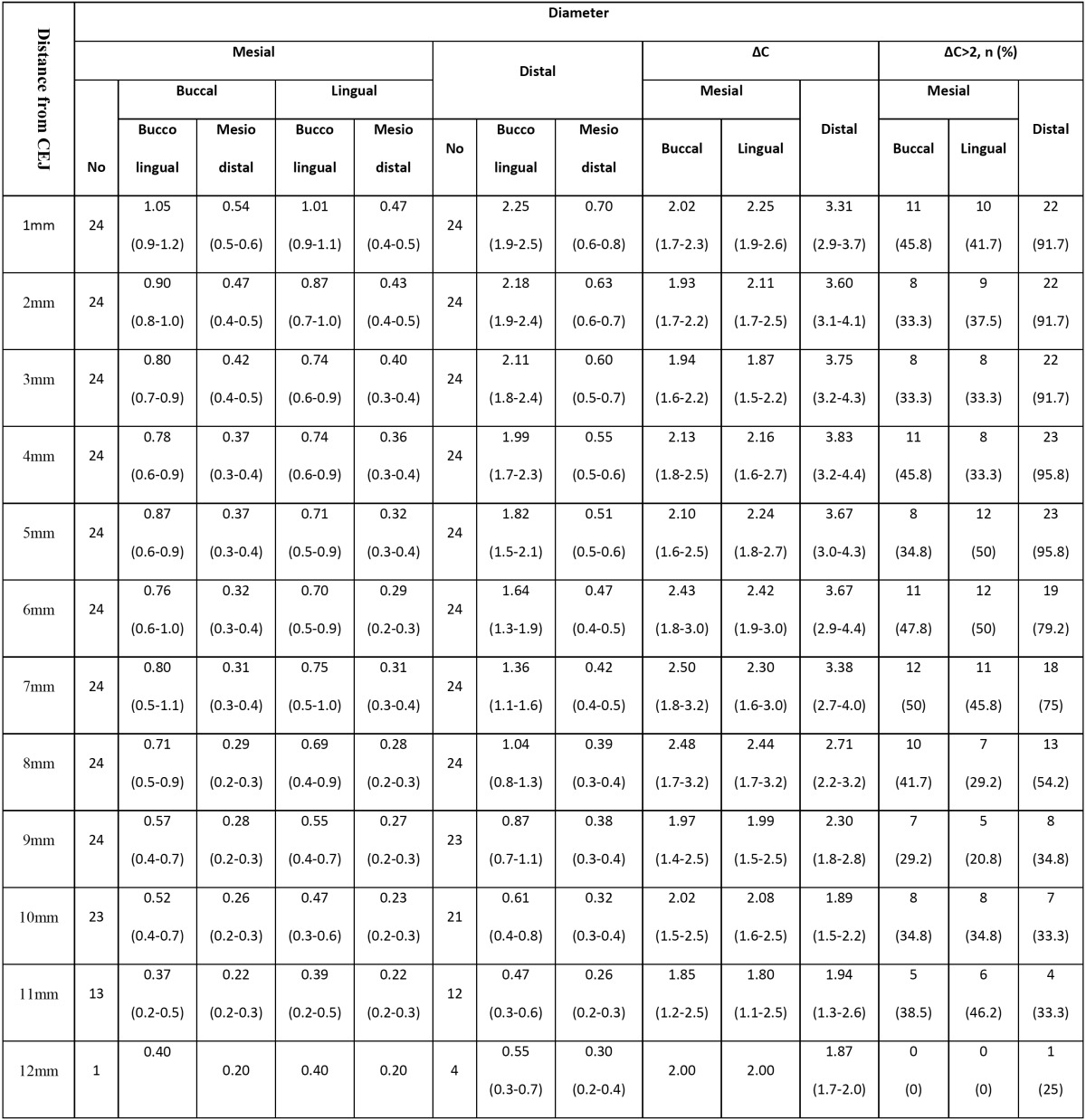
The mean (95% CI) canal diameter of the mesial and distal roots and distance from the furcation (mm).

 Table 4 shows the mean (±SD) canal taper; which was higher in the BL than MD dimension in the mesial and distal roots.

**Table 4:**
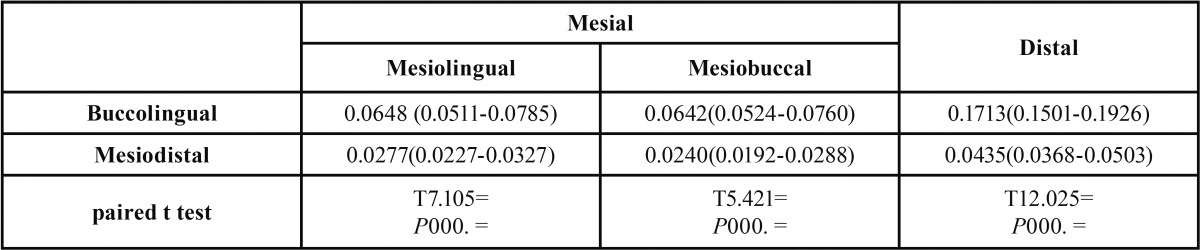
The mean (95% CI) canal taper in the mesial (ML and MB) and distal roots in BL and MD dimensions and the respective statistical results.

## Discussion

The results of this study showed that the ICC for the accuracy of linear measurement of radicular wall thickness was over 0.996, consistent with other studies (7). In other words, 3D and linear CBCT measurements were similar to the gold standard and thus, were highly accurate. The current study was conducted on human teeth and assessments were made in details; this was in contrast to most previous studies, which utilized skulls for evaluation of the accuracy of linear measurements using CBCT (8,9). Measurements made on the skulls have had a variable range of ICC from 0.995 to 1 (10). A previous study reported an ICC of 0.975 between CBCT and actual value in 22 linear measurements of anatomical landmarks in 23 skulls (11). Another study evaluated the validity of CBCT for measurement of human tooth length and width compared to caliper and reported no significant difference between the CBCT and caliper measurements (*P*=0.1145) (12). Another study evaluated the diagnostic accuracy of CBCT for measurement of tooth and root length in 7 pigs and found no significant difference between CBCT and caliper measurements with a mean error value less than 0.3mm (13). These results indicate the high diagnostic accuracy of CBCT for measurements and are in accord with our findings. On the other hand, in our study, the mean error of values measured by CBCT compared to the gold standard was 0.093mm; although this difference was not significant. This result is similar to the findings of some previous studies like the one evaluating the accuracy of linear measurements by NewTom QR DVT 9000, which reported that CBCT measurements had averagely 0.07mm difference with the actual values (14). Fatemitabar *et al.* reported that the mean differences varied from 0.37mm to 0.58mm for CBCT (Planmeca) and 0.37mm to 0.72mm for 64-channel CT (Siemens); although these values were higher than our results, the difference was not statistically significant (8). Pixel size plays an important role in the diagnostic accuracy of CBCT. Pixel size affects the spatial resolution. Pixel size and the consequently selected voxel size larger than the object size lead to partial volume averaging and errors in the diagnostic accuracy (15). In other words, if a precise section is made at the apical foramen, comparison of CBCT numbers with the gold standard sections seems necessary. It means that, in sections of the canal where the size of the respective area is less than 1/10 of a millimeter, the measured values may not have adequate accuracy. Further studies are required to better elucidate this issue.

In our study, after approving the diagnostic accuracy of CBCT, canal dimensions were measured. The results showed that in the mandibular first molars, distal root was shorter than the mesial root and the lingual furcation was farther from the CEJ than the buccal furcation. These results are similar to the findings of Gu *et al.*, who evaluated the morphology of mandibular first molars using μ-CT (16). Since the conventional 2D periapical radiography cannot provide the clinicians with adequate information about the buccal and lingual furcation sites, CBCT can be a good alternative for this purpose.

As seen in  Table 2, buccal and lingual canal walls in the mesial and distal roots of the mandibular first molars were thicker than the corresponding mesial and distal walls. In a previous study on 220 extracted teeth similar results were reported; although the researchers reported thicker lingual than buccal walls (17). In our study, the lingual wall was thicker in the majority of sections and this difference was statistically significant in the distal root (*P*<0.05) and not significant (*P*>0.5) in the mesial root.

Over-preparation of walls during cleaning and shaping can lead to immediate perforation of roots or their weakening and vertical fracture over time. Thus, it is recommended that at the end of root canal preparation, at least 1mm of dentin should remain around the root canal (18). This principle should be strictly followed by the clinicians in RCT. Another study evaluated the radicular wall thickness during post space preparation using periapical radiography and demonstrated that at different root canal preparation steps, periapical radiography overestimates the radicular wall thickness by 25% (19). Thus, a scientific consensus was reached that in RCT, the clinicians should not rely on periapical radiography alone. Our results showed that in the mesial root, the DB and DL wall thickness at 3mm distance from the furcation and the MB and ML wall thickness at 5mm distance from the furcation decrease to approximately 1mm. At 4mm distance from the furcation apically in the mesial wall and at 8mm distance from the furcation apically at the distal wall of the distal root this value approximated 1mm. These results indicate that in the clinical setting distal root should be preferred for post space preparation. These results are similar to the findings of a recently published systematic review (20).

The distal wall thickness in the mesial root and the mesial wall thickness in the distal root are usually smaller than that of other walls. Therefore, in endodontics, these areas are referred to as the danger zones (21). These areas are the thinnest and indicate that the canals are not centrally located in roots. Clinicians should consider the risk of strip perforation during canal preparation. Several microscopic studies have evaluated the thickness of dentin walls at the danger zones. One study reported the mean dentin thickness to be 1.119±0.273mm at the danger zone of mesial root of mandibular molars (22). However, this value was reported to be 1.05±0.33mm (23). In another study, this value was 1.2±0.3mm in the mesial and 1.98±0.3mm in the distal root (24). In our study, at 1 to 4mm distance from the furcation, canal wall thickness decreased from 1.23 to 0.89mm in the distal wall of mesial root and from 1.56 to 0.94mm in the mesial wall of the distal root. Such range of alterations covers the values reported in previous studies. However, the main advantage of our study was that we measured and reported the root dentin thickness at sections with regular 1mm intervals from the furcation apically.

Literature search yielded numerous studies on the prevalence of different root canal types and variations. However, studies on the effect of these configurations on the canal diameter and its cleaning and shaping are scarce (25,26). As seen in  Table 3, in some sections from the furcation towards the apex, values did not follow a regular sequential order; which is due to the anatomical variations and intracanal configurations. In our study, mesial root canals were Vertucci’s type IV in 18 and Vertucci’s type II in the remaining 6 teeth. If type II canals are excluded, the values follow a logical descending order. By taking into account this finding and using advanced imaging techniques like CBCT, the clinicians can employ necessary instruments and techniques based on individual anatomical variations and canal configurations.

Adequate root canal preparation is more difficult to achieve in oval compared to round canals. Use of large diameter endodontic instruments weakens the radicular mesial and distal walls while using small diameter instruments increases the risk of inadequate canal preparation particularly in the buccal and lingual walls. Previous studies have concluded that human root canals are mostly round in shape (27). However, according to some researchers, an oval canal is defined as a canal with large to small diameter ratio of over 2. If this ratio is smaller than 2, the canal is considered to be round (28). Our results demonstrated that the prevalence of oval canals in the distal root and at 5mm from the furcation was over 90%. This rate was <50% in the mesial root. Thus, in terms of the variability of canal shapes in our study, ML and MB roots were mostly round while distal roots were mostly oval. Recently, it has been recommended to use self-adjusting files (SAF) for canal preparation. It has been claimed that SAFs in contrast to NiTi files easily follow the canal shape. Thus, the original canal shape is preserved at the end of canal preparation phase (29).

According to the American National Standards Institute (ANSI) #58 for Hedstrom and ANSI #28 for K files (30), the taper of files per each millimeter of length should be 0.02mm. A noteworthy finding regarding the taper of MB and ML canals in our study was that the MD taper of these canals was 0.02 (0.027 in the MB and 0.024 in the ML canal); which was the exact same as the taper of endodontic instruments. Canal preparation strategy should precisely follow the unique anatomy of each canal. Thus, the same instruments or technique of preparation should not be applied to all root canals.

Collection of mandibular first molars that met the inclusion criteria of our study took so much time. Time and energy could have been saved if we had access to a tooth bank. Further studies are required to compare the diagnostic accuracy of CBCT with histological sections and µCT at the apical foramen. Also, similar studies are required on the anatomy and dimensional measurements of other teeth using CBCT.

## Conclusions

Within the limitations of this in-vitro study, the following conclusions were drawn:

CBCT has acceptable diagnostic accuracy for measurement of canal wall thickness.

In case of requiring the exact canal wall thickness values before or during an endodontic treatment, 3D imaging technique is recommended due to its optimal accuracy for endodontic measurements.

Our obtained results regarding the morphological and anatomical variations of mandibular first molars confirmed the results of previous studies. Moreover, this study was the first to accurately report the degree of taper of canals.

Considering all the above, use of advanced 3D imaging techniques is recommended for endodontic treatment particularly when precise information regarding the root canal anatomy and morphology is needed.
